# Supplementary material for: Patterns of Intron Gain and Loss in Fungi
Source: PLoS Biol. 2004 Nov 30;2(12):e422. doi: 10.1371/journal.pbio.0020422 (PMC532390; doi:10.1371/journal.pbio.0020422)
Supplement: Table S1 — Also available at http://genes.mit.edu/NielsenEtAl/. (4.3 MB ZIP). [file pbio.0020422.st001.zip › NielsenEtAl/html/823.html]

AN4353.1.NCU03372.1.MG02409.1.FG10853.1


```
 CLUSTAL W (1.82) Multiple Sequence Alignments - Introns Inserted


Sequence 1: NCU03372.1	458 aa
Sequence 2: FG10853.1	458 aa
Sequence 3: AN4353.1	458 aa
Sequence 4: MG02409.1	463 aa
Alignment Length: 463 aa
Number Identitical Residues: 286 aa
Alignment Score (without introns) 13085


MG02409.1 	MAPAKPKQPAYVLGVGMTQFIKPRGKVDYTELGFEAGVKAMLDARINYDEVDQGVACYCY
NCU03372.1	MAPRQ-KKPCYVLGVGMTKFIKPRGKVDYTELGFEAGVKAMLDAQISYDDVEQGIACYCY
FG10853.1 	MSSNK-KSPVYVLGVGMTKFIKPRGKVDYTELGFEAGVKALLDAQINYDDVDQGIACYCY
AN4353.1  	MGKKA-ASPAYVLGVGMTKFIKPRGKVDYNELGFEAGVKALLDAHINYDDVEQGVACYVY
          	*.     .* ********:**********.**********:***:*.**:*:**:*** *

MG02409.1 	GDSTCGQRVFYQFGMTQIPIVNVNNNCSTGSTGLAMARQLVGHGAADCVLVIGFEKMMPG
NCU03372.1	GDSTCGQRVFYQFGMTQIPIYNVNNNCSTGSTGLAMGRNFISSGAADCVLVVGFEKMQAG
FG10853.1 	GDSTCGQRVFYQFGMTQIPIYNVNNNCSTGSTGLNMARTLVEHGAAECVMVVGFEKMMPG
AN4353.1  	GDSTCGQRVFYQFGLTQIPIYNVNNNCSTGSTGLAMARTLVSHGAADCVMVVGFEKMSPG
          	**************:***** ************* *.* ::  ***:**:*:***** .*

MG02409.1 	SLQTFFQDRENPTGTSGLMMASTRGYEPKVPGAAQMFGNAGREYMEK~YGATLEDFAEIG
NCU03372.1	SLQSHFNDRENPLGTTYKMMATTRGVT-KAPGAAQMFGNAGREYMEK2YGAKPEDFAEIA
FG10853.1 	SLQSFFKDRESPTGTTVKMMADTRGIT-NSPGAAQMFGNAGREYMEK2YGATAEDFAEIA
AN4353.1  	SLQSVYNDRANPTGLFGMMMAETRGVT-NAPGAAQMFGNAGREYMEK2YGAKAEHFAEIA
          	***: ::** .* *    *** ***   : ***************** ***. *.****.

MG02409.1 	RVNHEHSARNPYSQFKDQYTLEQVMKSPMIHTPLTKLQCCPTSDGGAAAVVVSQAFLDAR
NCU03372.1	RINHAHSVNNPYSQFQDVYTLDQIMQSPKVHEPLTKLQCCPTSDGGAAAVLVSQEFLDAR
FG10853.1 	RINHAHSPNNPYSQFQDVYTKEQILQSPMIHEPLTKLQCCPTSDGGAAAIIVSEAFLNAR
AN4353.1  	RINHEHSKRNPYSQFQDEYTLEQVMKAPMIHEPLTKLQCCPTSDGAAAAIIVSQAFLDAR
          	*:** ** .******:* ** :*::::* :* *************.***::**: **:**

MG02409.1 	PELKANAIKIAGQCMATDGPELFSKSAIDLVGYGMGAHAGRVACAEAGVSPRDCQVCELH
NCU03372.1	PHLKDQAILIAGQCLATDAPSLFSKSAIDLMGWDMTQHAARVAMKEAGVKPSDFAVCELH
FG10853.1 	PHLREQAVEIAGQHLATDAPSLFSRSAIDLMGYEMTQRAMQEATKQAGISPRDVQVVEVH
AN4353.1  	PHLKDQAILIAGQQLATDNTTLYNRSSIDLMGFGMSRAACRAAVAEAGVNIKNIKVCELH
          	*.*: :*: **** :*** . *:.:*:***:*: *   * : *  :**:.  :  * *:*

MG02409.1 	DCFSANEMVTLDALGLADKGKAHELVRSGGITYPGGPGRTVVNPSGGLISKGHPLGATGL
NCU03372.1	DCFSANEMILIDALGFSEPGKAHEFVRAGNITYGG---KVVVNPSGGLISKGHPLGATGI
FG10853.1 	DCFSANEMVTIDALRLCDRGKAHELVRNGDITYGG---KYIINPSGGLISKGHPLGATGI
AN4353.1  	DCFSANEMITIDALELSEPGKAHEMVAKGDITYGG---KMVINPSGGLISKGHPLGATGL
          	********: :*** :.: *****:*  *.*** *   : ::*****************:

MG02409.1 	AQCAELVWHLRGWASNRAVPGTKNALQHNLGLGGAVVVTVYQRADGKEAPKLSEEEIIKA
NCU03372.1	AQCAELVWHLRGWANNRNVPHTKYALQHNLGLGGAAVVTVYKRADGKEAPVIDSATVGKL
FG10853.1 	AQCAELVWHLRGWANNRAAPNTRYCLQHNLGLGGAAVVTIYKRADGRAAPAVNSTMVGNR
AN4353.1  	AQCTELVWHLRGWANNRLVEGTDAALQHNLGLGGAVVVTVYKRADGKVAKPVSSADVAKI
          	***:**********.** .  *  .**********.***:*:****: *  :..  : : 

MG02409.1 	AGVGYNPAVQAKGFTAAQAKAVRSKEKSSEWALQDTLDKVQARF
NCU03372.1	SKLGYNPAVEAKGFTAEQVKAVRSREKVNEWALQDTMTKVEARF
FG10853.1 	NKLGYNPAVVAKGFTQEHVDMVRSKKARSEWALQDVENKVEARF
AN4353.1  	NGLGYNPAVEAKGFTAEQAKKVLSKTASDAWALADTQEKVIARF
          	  :****** *****  :.. * *:   . *** *.  ** ***
```
